# Disabled life expectancy among older Colombian men and women

**DOI:** 10.1371/journal.pone.0296638

**Published:** 2024-01-11

**Authors:** Margarita Osuna, Mateo Farina, Jennifer Ailshire

**Affiliations:** 1 Leonard Davis School of Gerontology, University of Southern California, Los Angeles, California, United States of America; 2 Department of Human Development and Family Science, Austin, Texas, United States of America; Drexel University, UNITED STATES

## Abstract

Colombia’s population is rapidly aging and older adults are living longer, however, we have limited information on the level of disability and number of years older Colombians spend with disability. We estimated age-and-gender specific ADL, IADL and mobility disability prevalence and disabled life expectancy (DLE) and to examined gender differences. Life tables came from the Colombian vital statistics and disability prevalence data came from the cross-sectional 2015 Colombia National Survey of Health, Well-being, and Aging. Disabled life expectancy (DLE) was calculated using Sullivan’s method. About one-third to one-half of remaining years will be spent with IADL or mobility disability. The remaining years of life spent with ADL was relatively low at younger ages, but by age 85, about half of remaining life will be spent with disability. Compared to men, women had higher levels of disability and are estimated to spend more years with disability. Gender differences in ADL did not emerge until ages 70 and older. Older Colombians, in particularly women, are estimated to live a significant proportion of their life with disability, particularly IADL and mobility disability. High levels of disability are concerning because the country lacks adequate infrastructure and has limited options for long term care.

## Introduction

There is a growing interest in understanding the health dynamics of older Colombians because the proportion of adults ages 60+ in Colombia has more than doubled in the past several decades–from about 4% in 1980 to 10% in 2020 [1]–and older adults are living longer. In 1993 life expectancy for a 60-year-old woman was 20.8 years [2], and according to the most recent estimates, life expectancy in 2019 would be of 25.3 years [3]. Older Colombian men have experienced greater gains in life expectancy, with years of remaining life at age 60 increasing from 14.9 years in 1993 [2] to 22.5 years in 2019 [3]. While increased longevity may potentially indicate improvements in health through lower mortality risk, it remains unclear if other health indicators show similar improvements. For example, a key question is whether the additional years are spent in good health. Understanding the health conditions of the older adult population is particularly important in a rapidly aging country like Colombia, since more time spent in poor health can contribute to increases in the costs of healthcare and social support services, and a greater need for long-term care [4, 5].

One of the major challenges with aging is increased risk of disability which can have a large impact on maintaining independence and quality of life among older adults [6, 7]. Older adults who have difficulty performing Activities of Daily Living (ADL), such as self-care, have increased daily care needs with assistance often coming from family members or paid caregivers [8]. Although there has been limited research on disability prevalence among the older population in Colombia, a study using nationally representative data on adults ages 60 and older found that around 15% of men and 25% of women have at least one ADL limitation [9]. However, disability increases significantly with each decade of age, and ADL prevalence estimates for the total population likely obscures large variation across age groups. For example, studies from other Latin American countries, such as Ecuador and Mexico, have shown that the prevalence of ADL disability tends to be much higher among those ages 80 and older than those in their 60s [10, 11].

Research has highlighted that instrumental activities of daily living (IADL) tend to precede the onset of ADL limitations [12, 13]; therefore, they are often more prevalent in the older adult population. IADLs include household chores and running errands, which can affect an older adult’s ability to age in place and remain independent [14, 15]. Studies in other countries of Latin America have shown that IADL prevalence increases with age and is higher among women [9, 10, 16, 17]. Among older Colombians, studies have reported a prevalence of limitations in IADLs of 35% for men and 40% for women [18].

Another commonly experienced disability among older adults is mobility disability. In the Colombian context, being able to walk several blocks and climb stairs, is key to living independently, especially since disability accommodations in the built environment are rare. Studies in other countries have highlighted that while mobility disability may not require the same level of care as ADL/IADL, it is essential for independent living and quality of life [19, 20]. Thus, mobility impairment is another important aspect of the disability experience of older Colombians. Using a measure of mobility disability that included limitations in walking and/or climbing, one study of Colombians ages 60 and older found that mobility disability prevalence was 25% for men and 40% for women [9]. Studies from other countries in Latin America have reported an even higher prevalence of mobility disability for men 23–36%, and similarly for women 32–36% [21]. Differences between these studies may be due to the greater number of mobility limitations (e.g., kneeling, crouching, etc.,) used in the studies. Mobility disability is likely much higher at older ages than younger ages, but prevalence has not been reported by age groups among older Colombians.

Gender differences in disability have been well-documented. Prior research has shown that women tend to be more likely to have disabling non-lethal conditions [22]. Women have a higher disability prevalence across several country contexts, including low-to-middle income countries [11, 23]. Across Latin American countries, including Colombia, data have shown that women have higher ADL, IADL and mobility disability prevalence than men [9–11].

While disability prevalence and mortality risk differ among Colombian men and women, research has yet to examine the extent of gender differences and the years of life spent with disability. Disabled life expectancy (DLE) is a well-established and broadly used summary measure of the number of years individuals are expected to live with a disability from a given age [24]. DLE is calculated with age-specific disability prevalence and mortality rates and is a useful measure to describe individual and population-level burden of disability [25, 26]. Additionally, DLE is a useful measure since combining this measurement with life expectancy researchers can estimate the proportion of life spent with a disability. Research in Latin America has shown that there is a gender gap where women live longer lives with more disability. A comparison of adults 60+ between seven capital cities in Latin America (Buenos Aires, Bridgetown, Sao Paulo, Santiago, Havana, Mexico City and Montevideo) found that women have higher DLE compared to men and that, although the gender gap was greatest at younger ages, it persisted into older ages [27]. To our knowledge, very few studies in Latin America have used nationally representative data on older adults to estimate DLE based on ADLs [10, 17, 28], and results from these studies indicate there is significant variation across Latin American countries. For example, at age 60 the proportion of life lived with ADL disability in Ecuador is almost two times greater that of Chile and about 50% greater than that found in Costa Rica. In all three countries women have a higher proportion of life spent with ADL disability (16.4% in Chile, 28.9% in Costa Rica and 32.7% in Ecuador) than men (11.3% in Chile, 20.0% in Costa Rica and 20.4% in Ecuador) [10, 28]. A study of DLE in IADL disability was done in Ecuador [10], and found that the proportion of DLE was higher among women (48.2%) compared to men (29%). No studies have determined DLE from mobility disability in Latin American countries and no studies have examined gender differences in DLE in ADL or IADL disability in Colombia.

Colombia presents a unique context to study disability and disabled life expectancy. Improvements in early life conditions and recent significant investments in human capital [29, 30], and infrastructure, have undoubtedly led to longer lives. Older adults in the country have also experienced unique historical hardships, such as heightened stress and health risks from a violent internal conflict that has lasted over five decades, that are associated with increased risk for disease [31–34]. Similarly, older Colombians may have suffered from poorer early life conditions such as poverty, malnutrition, lack of access to healthcare, among others, which can also contribute to a higher risk of disability. Research in other countries of Latin America have highlighted that early life childhood conditions, specifically poverty, increase the risk of disabled life expectancy among older adults [35]. Colombia has a high rate of poverty among older adults [36], and given lack of opportunities for upward economic mobility in the country [37] it is likely that these adults have lived in impoverished conditions their entire lives. Research has found that older Colombians who live in states with higher rates of poverty have a higher prevalence of ADLs [38]. Colombia also lacks much of the infrastructure needed to support older adults with functional limitations (e.g., accessible sidewalks, ramps, and public transport), which can accelerate the disablement process [39–41]. Given the hardships that older Colombians have been exposed to throughout their lives, and the potential consequences for old age disability burden, as well as recent increases in older age life expectancy, we might expect older Colombians to have relatively high DLE.

In this study, we use nationally representative data to estimate disability life expectancy for older Colombian men and women. Our study has two main objectives: 1) describe disability prevalence in the population by age and gender; and 2) determine DLE, or the proportion of remaining years of life spent with disability, for men and women at different ages. We focus on three different measures of disability: ADL, IADL, and mobility limitations. We examine DLE separately by gender since women live longer than men [42], can have worse health conditions [43, 44] and tend to have more disabling conditions [22].

## Methods

### Data

Calculations of disabled life expectancy require information on age-specific mortality from life tables and survey-based disability prevalence. We use life tables from the Colombian National Administrative Department of Statistics (DANE), which compiles vital statistics and conducts the national census of population for Colombia. and uses this information to calculate gender- and age-specific mortality data as well as total life expectancies. We used publicly available life tables for 2015 that DANE constructed based on annual death counts in 2015 and 2018 census-adjusted population counts [45]. We use the 2015 Colombia National Survey of Health, Well-being, and Aging “Encuesta Nacional de Salud, Bienestar y Envejecimiento” (SABE-COL) to determine disability prevalence. SABE-COL is the first nationally representative survey of older adults ages 60 and older in Colombia and was designed to obtain detailed information on the social, economic, and health conditions of older adults. Face-to-face interviews were conducted with all respondents or their proxies. Of the 23,694 respondents in SABE, we excluded 68 cases that had missing data on disability status. Our analytic sample consisted of 23,619 respondents.

The 2015 SABE Colombia was collected by the Colombian Ministry of Health. The databases were fully anonymized by the Colombian Ministry of Health before they were made publicly available for research purposes. The Institutional Review Board of the University of Southern California approved the use of the data and its analysis for the authors of the paper.

### Measures

Disabled life expectancy (DLE) is the expected number of years lived with a disability. We examine three different dimensions of disability: limitations in activities of daily living (ADL), limitations in instrumental activities of daily living (IADL), and mobility limitations.

#### Activities of daily living

Respondents were asked if they currently needed help performing six activities of daily living: eating, dressing, bathing, toileting, getting out of bed, and walking across a room. For each of the activities, respondents or their proxies indicated if the respondent: (1) could do the activity independently (including if they relied on a cane or walker), (2) needed help to do the activity, or (3) were unable to do the activity. We constructed a dichotomous measure representing a limitation in any ADLs, where 0 indicated independence doing all activities and 1 indicated difficulty or inability to do any of the activities.

#### Instrumental activities of daily living

Respondents were asked if they currently had any difficulties or needed help performing four instrumental activities of daily living: grocery shopping, taking responsibility for their own medications, using public transportation, and using the phone. For each of the activities, respondents or their proxies indicated if the respondent: (1) could perform the activity without difficulty or need of help, (2) could do the activity without help, but with difficulty, (3) needed help, (4) was unable to do it and (5) does not apply. We constructed a dichotomous measure for limitation in any IADLs, where 0 indicated independence in doing all the activities and 1 indicated difficulty or inability to do any of the activities. Respondents who answered “Does not apply” were coded as 0 (independence in performing activity) since their response does not indicate difficulty with or inability to do the activity. Prior work has shown that men are more likely to select “does not apply” on activities that tend to be performed by women, such as cooking, and it has been suggested that coding these responses as missing could result in gender bias in IADL prevalence [46].

#### Mobility difficulty

Respondents were asked about difficulty walking 400 meters (approximately 5 blocks) or climbing stairs. Walking difficulty had the following response options: (1) has no difficulty, (2) has some difficulty, (3) has a lot of difficulty and (4) is unable to perform the activity.

The response options for difficulty climbing stairs were: (1) can go up and down the stairs independently, (2) needs help going up and down the stairs, and (3) is not able to go up or down the stairs. We constructed a dichotomous measure for mobility disability where 0 indicated independence in both walking (i.e., reported no difficulty) and climbing and 1 indicated difficulty with or inability to do either of these activities.

### Other covariates

We also include age, which we categorized into 5-year age groups (60–64, 65–69, 70–74, 75–79,80–84,85+) and gender.

### Analytic strategy

DLE reflects the average number of years a person is expected to live with a disability at a specified age. We combine information from gender-specific 5-year abridged-life tables with gender and age interval-specific disability prevalence. We calculated prevalence for IADL, ADL and mobility disability by age group and gender. Prevalence estimates were weighted using survey sample weights to ensure estimates reflect the distribution of the Colombian older adult population. We use the Sullivan method to calculate DLE [24, 27]. First, we calculated the person years lived without disability for each age interval. Next, we summed the total number of person years lived without disability after age x for each age interval. To determine disability-free life expectancy we divide the number of person years lived without disability by the number of people surviving to each age interval (the number surviving to each age interval is taken from the life table synthetic cohort as of age 60). Finally, in order to determine disabled-life expectancy we subtract the number of disability-free years from total life expectancy for each age interval [24].

We also calculated standard errors of DLE by estimating, for each age interval, the sum of the product of the square of person years lived and the variance of the prevalence rates. Using standard errors, we calculate 95% confidence intervals by multiplying DLE at *age x* times the standard error of DLE ±1.96.


$$Confidence\;Interval={DLE}_{x}\times (\pm 1.96\times Standar\;Error\;of\;DLE)$$

More detail on how these calculations can be performed are found on the Health Expectancy Calculation by the Sullivan method guide [24, 47]. We then calculated z scores to determine if there were statistically significant differences between genders. Z statistics were calculated by dividing the difference in DLE between males and females over the sum of standard error of males + females [24]. Each calculation was done for the three measures of disability: ADL, IADL and mobility disability. These life tables also were used to calculate the proportion of years spent with disability.

## Results

**Fig 1:**
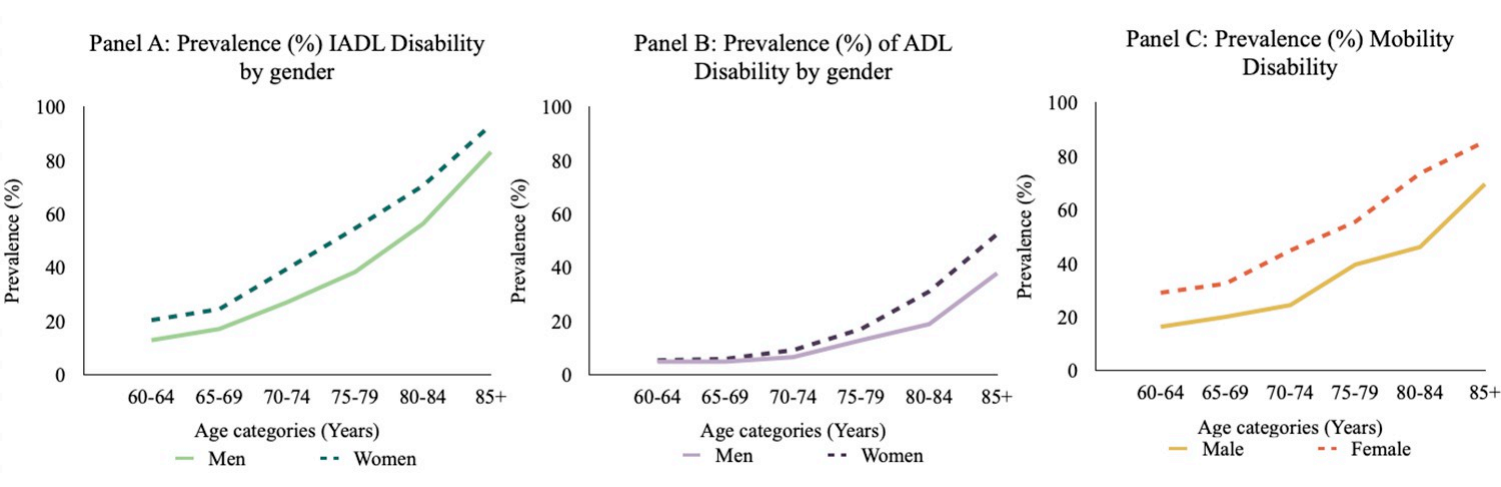
Prevalence of disability by age and gender.

Fig 1 shows the prevalence of ADL, IADL and mobility disability, for both men and women, across age groups. The prevalence of IADL disability (Panel A) is about 13% for men and 20% for women at ages 60–64 but increases sharply with age to 83% and 93% of 85+ men and women, respectively. ADL disability prevalence (Panel B) also increases with age, but not as steeply as observed with IADLs. At ages 60–64 only about 5% of men and women have an ADL limitation, but by ages 85+ one-third of men and over half of women have ADL disability. Mobility limitations also increase with age, from about 16% at ages 60–64 to nearly 70% at ages 85+ for men and from 30% to 85% for women.

Table 1 shows the prevalence estimates (and 95% confidence intervals) for each measure of disability for men and women by age groups, as well the gender difference in prevalence and tests of the gender difference. Women had higher IADL prevalence than men at all ages (p < .01), with the largest gender gap occurring at ages 75–84. Gender differences in ADL prevalence begin to emerge at age 70 (p < .01), with women reporting more ADL disability than men. Women also had higher prevalence of mobility disability at all ages (p < .01). Among all disability measures, the largest gender gap was found for mobility disability.

**Table 1:** Prevalence of IADL, ADL, and mobility disability by age and gender with associated 95% confidence intervals and tests of gender differences, SABE-Colombia 2015.

		Men		Women		Gender △	
	Age	%	95% CI	%	95% CI		
IADL	60–64	12.7	(10.5, 15.2)	20.3	(17.2, 23.9)	7.6	*
	65–69	16.9	(13.6, 20.6)	24.3	(20.9, 28.1)	7.4	*
	70–74	27.0	(22.3, 32.2)	39.8	(35.4, 44.3)	12.8	*
	75–79	38.3	(32.4, 44.6)	54.5	(49.0, 59.9)	16.2	*
	80–84	56.2	(48.8, 63.4)	70.4	(64.8, 75.5)	14.2	*
	85+	83.0	(77.2, 87.6)	93.1	(90.4, 95.0)	10.1	*
ADL	60–64	4.7	(3.2, 6.9)	5.2	(3.4, 8.0)	0.5	
	65–69	4.7	(3.4, 6.4)	5.7	(4.2, 7.8)	1.0	
	70–74	6.4	(4.9, 8.4)	9.0	(6.9, 11.6)	2.6	*
	75–79	12.7	(8.0, 19.6)	16.9	(12.2, 23)	4.2	*
	80–84	18.7	(13.9, 24.6)	30.9	(26.0, 36.2)	12.2	*
	85+	37.7	(29.5, 46.7)	52.3	(44.9, 59.6)	14.6	*
Mobility	60–64	16.2	(13.0, 20.1)	28.9	(25.3, 32.7)	12.7	*
	65–69	19.9	(16.3, 23.9)	32.3	(28.6, 36.3)	12.4	*
	70–74	24.3	(20.3, 28.7)	44.7	(40.1, 49.3)	20.4	*
	75–79	39.4	(33.3, 45.9)	55.4	(49.8, 60.7)	16.0	*
	80–84	46.0	(38.7, 53.5)	73.5	(69.0, 77.5)	27.5	*
	85+	69.5	(61.4, 76.6)	85.4	(80.7, 89.1)	15.9	*

Note: All prevalence estimates are weighted. Gender differences were calculated by subtracting prevalence for men from prevalence for women, with higher values indicating higher prevalence in women. Gender differences were determined from two-tailed z-tests of equality of proportions (* p < .01).

Table 2 contains DLE estimates (and 95% confidence intervals) for each measure of disability for men and women by age groups, in addition to tests of the gender difference. Overall men had the highest DLE in IADLs followed by mobility and ADLs disability. Overall, women had a higher DLE in mobility followed by IADLs and ADLs. Gender differences were observed for all measures however, the largest gender difference was found in mobility disability. Gender differences decreased from 5.33 years difference at ages 60–64 to 1.53 years difference at ages 85+. We tested gender differences using a z-statistic test and all p values were < .001.

**Table 2:** Disabled life expectancy (DLE) by age and gender with associated 95% confidence intervals and tests for gender difference, SABE-Colombia 2015.

	Age	Men		Women		Gender △
		DLE	(95% CI)	DLE	(95% CI)	
IADLs	60–64	6.69	(6.52, 6.86)	10.94	(10.76, 11.11)	4.24
	65–69	6.52	(6.35, 6.69)	10.36	(10.19, 10.53)	3.84
	70–74	6.39	(6.22, 6.56)	9.81	(9.65, 9.98)	3.42
	75–79	6.15	(5.97, 6.33)	8.92	(8.77, 9.07)	2.77
	80–84	5.88	(5.70, 6.07)	7.83	(7.69, 7.96)	1.94
	85+	5.42	(5.23, 5.60)	6.67	(6.55, 6.78)	1.25
ADLs	60–64	2.44	(2.30, 2.57)	4.14	(3.99, 4.30)	1.71
	65–69	2.37	(2.23, 2.51)	4.05	(3.89, 4.21)	1.68
	70–74	2.40	(2.26, 2.55)	4.03	(3.86, 4.19)	1.63
	75–79	2.52	(2.36, 2.68)	4.08	(3.90, 4.26)	1.56
	80–84	2.55	(2.36, 2.74)	4.04	(3.84, 4.23)	1.49
	85+	2.69	(2.46, 2.93)	3.77	(3.55, 4.00)	1.08
Mobility	60–64	6.40	(11.91, 6.58)	11.73	(11.54, 11.94)	5.33
	65–69	6.03	(10.93, 6.2)	10.75	(10.58, 10.96)	4.73
	70–74	5.69	(10.03, 5.87)	9.86	(9.68, 10.05)	4.17
	75–79	5.46	(8.87, 5.65)	8.71	(8.55, 8.90)	3.25
	80–84	4.91	(7.71, 5.11)	7.55	(7.40, 7.73)	2.64
	85+	4.59	(6.27, 4.81)	6.11	(5.96, 6.30)	1.53

Note: All prevalence estimates are weighted. Gender differences were calculated by subtracting DLE for women from DLE from men, with higher values indicating higher DLE in women. Gender differences were determined using a z-statistic test and all p values were < .001.

**Fig 2:**
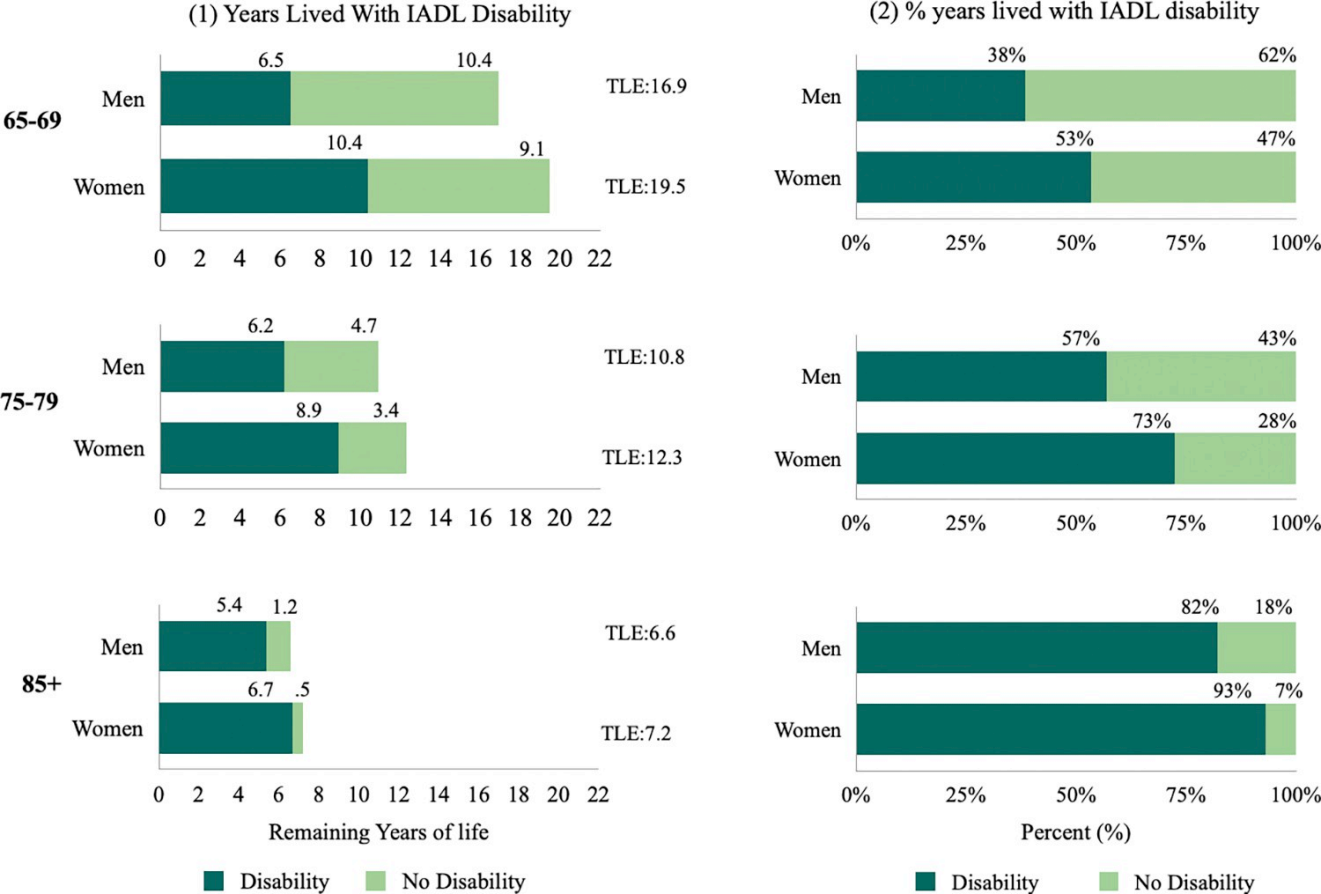
Total life expectancy (TLE), disabled life expectancy (DLE) and proportion of life with IADL disability by age for men and women, SABE-Colombia 2015. *Note: TLE: Total Life Expectancy.

**Fig 3:**
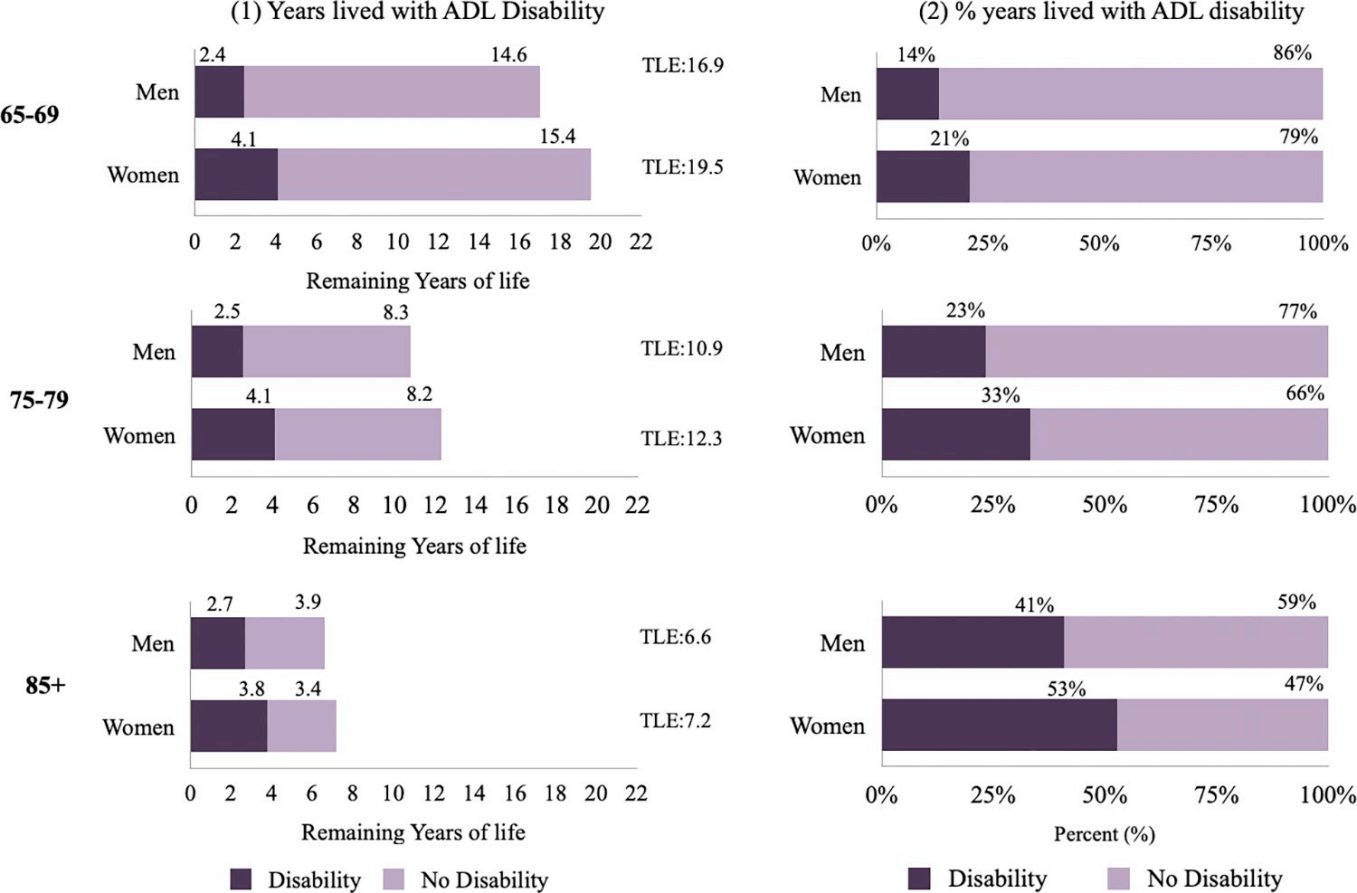
Total life expectancy (TLE), disabled life expectancy (DLE) and proportion of life with ADL disability by age for men and women, SABE-Colombia 2015. *Note: TLE: Total Life Expectancy.

**Fig 4:**
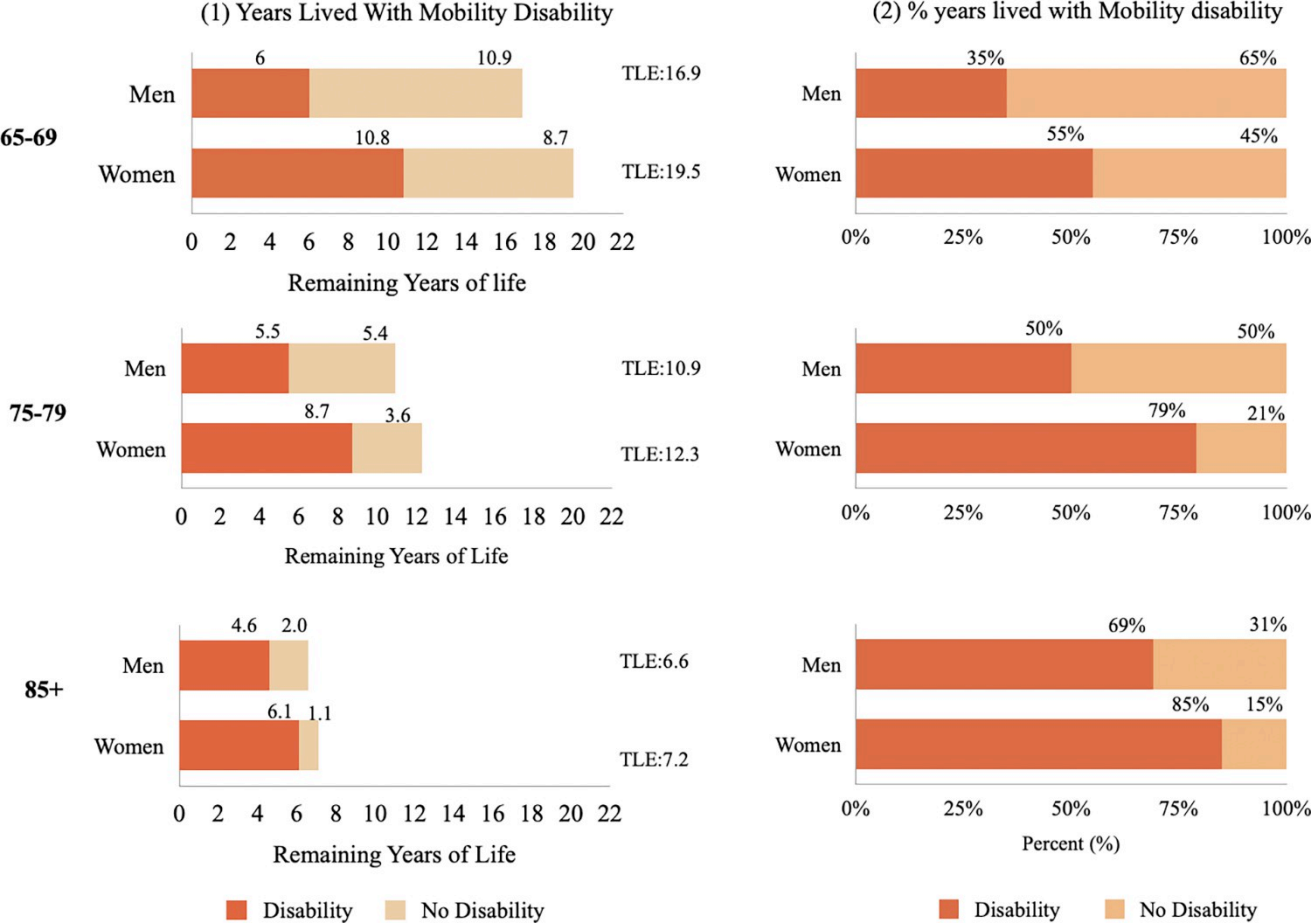
Total life expectancy (TLE), disabled life expectancy (DLE) and proportion of life with mobility disability by age for men and women, SABE-Colombia 2015. *Note: TLE: Total Life Expectancy.

Figs 2–4 show disabled life expectancy and the proportion of remaining life spent with disability for three age groups and by gender (S1 Table reports total life expectancy and proportion of life spent with a disability, as well as confidence intervals and p values). The figs on the left show total remaining life expectancy (TLE), which is represented by the length of bar, separated into the number of years lived with (dark area) and without (light area) disability. The figs on the right show the proportion of life spent with disability. Fig 2 shows that at ages 65–69, women’s remaining life expectancy is 19.5 years and are expected to live 53.3% of their remaining life with IADL disability. Men on the other hand, have 16.9 remaining years of life at 65–69 and will spend 38.4% of that time with IADL disability. Fig 3 shows that at age 65–69, women are expected to live 20.8% of their remaining life with ADL disability and men are expected to life 14.0% of their remaining years with ADL disability Fig 4 shows that at ages 65–69, women are expected to live 55.0% of their remaining life with mobility disability, while men at the same ages are expected to live 35.0% of their remaining years with mobility disability. The expected proportion of life lived with disability increases with age for both men and women, decreases at the oldest ages for IADL and mobility disability, and increases for ADL disability.

## Discussion

This study presents new estimates of disability prevalence and disabled life expectancy for men and women ages 60 and older in Colombia. IADL and mobility disability rose steeply with age among Colombian men and women starting at age 60, with increases in ADL disability observed from age 75. Compared to men, women have higher prevalence of IADL and mobility disability at all ages, and higher prevalence of ADL disability beginning around age 70. Among Colombians in their 60s, about one-third (men) to one-half (women) of remaining life will be spent with an IADL or mobility disability. By age 85 nearly all remaining life will be spent with IADL disability and well over half will be spent with mobility disability. Because ADL prevalence was relatively low at younger ages, only 14% (men) to 21% (women) of remaining life is expected to be spent with severe disability. However, by age 85 about one-half of remaining life is expected to be spent with disability. Importantly, because women nearly always have higher prevalence of disability than men, as well as longer life expectancy, they are expected to disproportionately spend more years of life with disability.

Our disability prevalence estimates suggest Colombian older adults may experience less disability than older adults in other Latin American countries with similar social and economic contexts. In Colombia, older adults ages 60–64 have an IADL disability prevalence of 13% for men and 20% for women. By comparison, a study reporting IADL prevalence in neighboring Ecuador, a country similar to Colombia in geography, built environment, and socio-political development, found similar IADL prevalence for men (13%), but higher prevalence for women (29%) [10]. At ages 80–84 IADL prevalence among Colombian women was 70%, compared to 73% among Ecuadorian women ages 80+ and was 56% among Colombian men, compared to 47% among Ecuadorian men [10]. The observed variations in IADL limitations between women for these two countries might stem from the age differences in reporting disability prevalence, since in Ecuador the authors include all adults ages 80 and older whereas the numbers reported for Colombia are for a younger group of adults ages 80–84.

With respect to ADL limitation, which reflects more severe disability, Colombians have substantially lower prevalence compared to other countries. At age 60–64, ADL prevalence was about 5% for Colombian men and women, which is much lower than the 14% and 24% ADL prevalence reported for men and women, respectively, in Ecuador [10]. Older Colombians also have lower ADL prevalence compared with Mexico, where prevalence at ages 65–69 is 10% for men and 15% for women [44]. Lower ADL disability prevalence in Colombia is observed at all ages when compared to other studies. It is possible that older Colombians experience lower IADL and ADL disability prevalence universal healthcare access, even though studies have highlighted that there are many limitations in the healthcare system [48, 49] it is possible that preventative measures and interventions reduce prevalence of disability.

Less is known about mobility disability in Latin America, which makes it challenging to determine if our estimates are high or low for the region. For our total sample, the prevalence of mobility disability was 21% for men and 43% for women. According to prevalence estimates published for seven capital cities of Latin America (Buenos Aires, Bridgetown, Sao Paulo, Santiago, Havana, Mexico City and Montevideo) mobility disability prevalence ranged from 23–36% among men and 32–36% among women [21]. By comparison, we found a mobility disability prevalence of 21% for men and 43% for women ages 60 and older in Colombia. However, our estimates are based on a nationally representative sample, while the estimates done by Alvarado et al [21] are from highly urbanized cities and likely underestimate disability due to the exclusion of poorer and more rural populations. On the other hand, our estimates of mobility disability may be more conservative, because we only had two measures of mobility (walking and climbing), whereas Alvarado et al [21] included five measures. It is possible that some of the differences we find in our results are due to the walking culture that exists in Colombia, it is very common to walk to get around in the country, and it might help older adults stay more mobile and have fewer movement problems than in other countries.

While disability prevalence provides a snapshot of population-level burden, population health scientists and demographers have often relied on disabled life expectancy measures to estimate DLE which is a comprehensive measure that summarizes the life expectancy and disability experienced by a real population [24, 26, 50]. Comparing our DLE with IADL results with Ecuador, we find similarities in the proportion of life spent with disability. For example, Ecuadorian women ages 60–64 will spend 48% of remaining life with an IADL limitations [10], compared to 46% of Colombian women at the same age. These patterns also hold for men in both countries. Similarly, at older ages, (ages 80+ for Ecuador and 80–84 in Colombia); women spend a similar proportion of life with IADL disability (Ecuadorian women 73% and Colombian women 70%) [10]. Whereas Colombian men spend a larger proportion of their life with disability (47% in Ecuador vs. 56% in Colombia). DLE and the proportion of life spent with disability may be higher at older ages due to a combination of higher prevalence and longer lives. Although longer lives are a sign of success for a country, our results indicate that a vast majority of older adults ages 80+ will have difficulty preforming at least one IADL and, that women have a higher disability burden. Similar findings have been discussed in low-and-high-income countries [10, 51]. These increases in life expectancy require additional research to understand under what health conditions older adults are aging [51, 52].

With respect to ADLs, we find that Colombian men and women have a lower DLE than most other Latin American countries. The proportion of life spent with ADL disability for women ages 60–64, was 33% in Ecuador [10], and at age 60 it was 16% in Chile and 22% in Costa Rica [28] which is higher than the proportion of life with ADL for Colombian women (11%) at the same age. We found a similar pattern for men; at the same ages, men in Ecuador (23%) and Costa Rica (15%) spent a higher proportion of life with disability than Colombian men (12%), however, men in Chile (11%), spent a lower proportion of life with ADL disability [10, 28]. Additionally, we can compare our results with Mexico and Ecuador for ages 65–69. At these ages, the proportion of life spent with ADL disability for Colombian women was 22%, whereas it was lower in Mexico (15%) [44] and higher in Ecuador (35%) [10]. At the same ages, the proportion of life spent with ADL disability was 14% for Colombian men, which is higher than in Mexico (9%) [44] but lower than in Ecuador (25%) [10]. These cross-national differences indicate that the proportion of life spent with ADL disability among older adults in Colombia was in between Mexico and Ecuador. Very few studies have data on the proportion of life spent with ADL disability among those ages 80+. However, we know that in Ecuador, women aged 80+ spend 50.4% [10] of their life with ADL disability, similar to what we found at ages 80–84 in Colombia (43%).Whereas Ecuadorian men ages 80+ [10] have a lower proportion of life spent with disability (34.2%) compared to Colombian men ages 80–84 (41%). This can be an indicator that those older women spend a larger proportion of their life a with ADL disability.

Our estimates of DLE for ADLs in Colombia are comparable to those found in high-income countries such as Spain and the United States [28, 53]. For example, at ages 60, Spanish women (DLE: 4.89) and men (DLE: 2.10) [28], have slightly higher DLE than Colombian women (DLE: 4.14) and men (DLE:2.44 years). Similarly, in the United States at ages 65–69, women (DLE: 4.99 years) and men (DLE: 3.1) [53] have a slightly higher DLE than Colombian women (DLE: 4.05) and men (DLE: 2.37). However, at ages 85+, US men had a lower DLE (DLE: 2.26) than Colombian men (DLE: 2.69), whereas US women had a higher DLE (DLE: 3.87) than Colombian women (3.77 years) [53]. Although years spent with ADL disability in Colombia are similar to those of high-income countries, the proportion of life that will be lived with disability is grater among Colombian older adults because their life expectancy is lower than in the United States and Spain.

Differences in DLE across Latin American countries may be driven by either difference in disability prevalence or mortality regimes that create differences in total life expectancy. Prior research in Latin America has shown large variations in life expectancy across countries [54–56]. These large variations of life expectancy are most likely indicative of significant variations in mortality rates across ages. However, in countries that are most similar to Colombia in their political, economic and social context, life expectancies [3] during 2015 are quite similar. Such as Ecuador (males: 21.4; females 23.6), Chile (males: 21.6; females 25.4) and Costa Rica (males: 23.3; females 26.4). This, in turn, may indicate that mortality schedules are similar across these contexts. Therefore, differences in DLE may be attributed more to disability rates–whether it is a difference in measurement or actual differences in underlying restrictions. Future research should examine the age schedule of mortality and the operationalization of disability across Latin American countries to better understand inequality in the region and determine how both processes to combine to shape the health of older adults across countries.

While DLE provides the number of years of which an older adult may live disabled, proportion of life spent with disease allows researchers to understand how disability impacts the number of years a person lives. A large proportion of Colombian older adults’ lives is expected to be spent with mobility disability. This is worrisome given that walking and climbing are a central to daily activities and the social lives of Colombians. The high proportion of life spent with a mobility disability may reflect underlying bodily impairment but could also reflect environmental factors, such as difficult walking conditions (e.g., uneven sidewalks, dirt roads), steep inclines, or lack of accessibility (e.g., ramps, handrails). Lack of infrastructure can make it difficult for older adults to retain their independence [57, 58]. In a country like Colombia, that has poor infrastructure and difficult environmental conditions (e.g., uneven sidewalks, steep heals, unpaved roads) older adults may face challenges in maintaining social interactions and independence [59–62]. For example, other studies in the country have highlighted that older adults that live in areas where there are better environment characteristics (Street crossings, low noise, low perceived danger of accident with traffic and an overall feeling of safety) have been associated with higher quality of life [59] and more time spent walking [60]. Although, mobility disability can be reduced if the older adult has access to assistive devices which may allow them to preform activities that were once difficult [63], navigating an environment with poor conditions may make it harder for older adults to have mobility ability.

We found large gender inequalities in disability prevalence and DLE consistent with prior research highlighting women’s disability disadvantage [3, 28, 64, 65]. Our results indicate that the largest gender difference in disability prevalence was for mobility at ages 80–84, where women’s disability prevalence was 27 percent points higher than men’s. Similarly, the largest gender difference in DLE was for mobility, at ages 60–64, where woman would spend a larger proportion of their remaining years with disability (55% of years lived with mobility disability) compared to men (35% of years lived with mobility disability). The gender disparity in DLE can stem from a combination of increased disability prevalence and life expectancy. We find that women live longer lives and spend a higher proportion of life with disability. This larger proportion of disabled life can be explained through the “male-female health-survival paradox” [17, 22, 65–67] where it is suggested that a combination of dimensions (biological differences, cultural factors, and risk behaviors) lead to women living longer but with worse health conditions. However, many studies in Latin America have highlighted that women have fewer educational opportunities, greater economic insecurity, poorer access to pensions, less access to social mobility and lower qualified employments. These cumulative disadvantages in gender exposures may lead to a greater rate of disability among women [17, 21, 28, 67, 68]. Taking this into account, future research should consider which factors contribute to Colombian women’s greater risk of disability by specifically investigated how the disablement process may be gendered.

This study has some limitations. First, given that the data that we are using is cross sectional, we could not take into account transitions across disabled states (i.e., recovery from disability). Therefore, our analysis is relying on a strong assumption that once in a disabled state the individual continues in that state. There are currently no population-based longitudinal surveys of older adults in Colombia from which disability transitions can be calculated. Additionally, SABE-Colombia surveyed only community-dwelling older adults and thus did not include the institutionalized older adult population, who tend to have the greatest care needs. However, institutionalization is very rare among older Colombians [4, 5] as few public options exist and private nursing homes are generally unaffordable. Another limitation is that we only had two measures of mobility disability: walking and climbing, whereas other studies had kneeling, crouching, lifting a heavy object, etc. These two measures may not capture disability severity to its fullest, albeit these are the most important measures in the Colombian context because walking and climbing are crucial to access different forms of transportation, social interactions and are useful to maintain independence. Given this, it is possible that our estimates may be somewhat more conservative than the true prevalence of disability in the population. Lastly, when we calculated our IADL measure we did considered that “does not apply” response option as not having a limitation, it is possible that this has affected our results, although literature has shown that men are more likely to select “does not apply” on activities that tend to be performed by women [46]. We checked for DLE differences including or excluding “does not apply” and found minimal differences, therefore we decided to follow what is suggested in the literature and code “does not apply” as not having a disability.

This study uses high quality data on disability and mortality that has recently become available for Colombia to give novel estimates of disabled life expectancy. Underreporting of mortality is a concern in some areas of Colombia, but we use data that have been corrected for mortality underreporting by the national statistical agency (DANE). Disability prevalence estimates come from the first nationally representative study of Colombian older adults, allowing us to calculate DLE estimates at the national level, than just major cities, as has been done in previous research. Another contribution of this study is that we examined multiple dimensions of disability, which provide a comprehensive understanding of the disability burden in Colombia. Overall our findings allow us to better understand under what health conditions older adults are aging.

## Supporting information

S1 Checklist STROBE statement—checklist of items that should be included in reports of observational studies. (DOCX)

S1 Table Years lived with and without disability, proportion of years disabled and life expectancy for both men and women by age categories. (DOCX)
