# Supplementary material for: Disabled life expectancy among older Colombian men and women
Source: PLoS One. 2024 Jan 11;19(1):e0296638. doi: 10.1371/journal.pone.0296638 (PMC10783758; doi:10.1371/journal.pone.0296638)
Supplement: S1 Checklist — (DOCX) [file pone.0296638.s001.docx]

STROBE Statement—checklist of items that should be included in reports of observational studies

|  | Item No. | Recommendation | Page  No. | Relevant text from manuscript |
| --- | --- | --- | --- | --- |
| **Title and abstract** | 1 | (*a*) Indicate the study’s design with a commonly used term in the title or the abstract | 2 | Cross-sectional 2015 Colombia National Survey of Health, Well-being, and Aging |
|  |  | (*b*) Provide in the abstract an informative and balanced summary of what was done and what was found | 2 | Older Colombians, in particularly women, are estimated to live a significant proportion of their life with disability, particularly IADL and mobility disability |
| Introduction | | | |  |
| Background/rationale | 2 | Explain the scientific background and rationale for the investigation being reported | 6 | No studies have determined DLE from mobility disability in Latin American countries and no studies have examined gender differences in DLE in ADL or IADL disability in Colombia. |
| Objectives | 3 | State specific objectives, including any prespecified hypotheses | 7 | Our study has two main objectives: 1) describe disability prevalence in the population by age and gender; and 2) determine DLE, or the proportion of remaining years of life spent with disability, for men and women at different ages. |
| Methods | | | |  |
| Study design | 4 | Present key elements of study design early in the paper | 7 | We examine DLE separately by gender since women live longer than men, can have worse health conditions and tend to have more disabling conditions. |
| Setting | 5 | Describe the setting, locations, and relevant dates, including periods of recruitment, exposure, follow-up, and data collection | 7 | We obtained age-specific mortality data for men and women from the 2015 vital statistics. We calculate disability prevalence using data from the 2015 Colombia National Survey of Health |
| Participants | 6 | (*a*) *Cohort study*—Give the eligibility criteria, and the sources and methods of selection of participants. Describe methods of follow-up  *Case-control study*—Give the eligibility criteria, and the sources and methods of case ascertainment and control selection. Give the rationale for the choice of cases and controls  *Cross-sectional study*—Give the eligibility criteria, and the sources and methods of selection of participants | 8 | Of the 23,694 respondents in SABE, we excluded 68 cases that had missing data on disability status. Our analytic sample consisted of 23,619 respondents. |
|  |  | (*b*) *Cohort study*—For matched studies, give matching criteria and number of exposed and unexposed  *Case-control study*—For matched studies, give matching criteria and the number of controls per case | NA | NA |
| Variables | 7 | Clearly define all outcomes, exposures, predictors, potential confounders, and effect modifiers. Give diagnostic criteria, if applicable | 8 | Measures: IADLs, ADLs, Mobility Disability |
| Data sources/ measurement | 8* | For each variable of interest, give sources of data and details of methods of assessment (measurement). Describe comparability of assessment methods if there is more than one group | *8/10* | Detailed description of each measure, how it was constructed and how it was measured. |
| Bias | 9 | Describe any efforts to address potential sources of bias | 9 | Prior work has shown that men are more likely to select “does not apply” on activities that tend to be performed by women, such as cooking, and it has been suggested that coding these responses as missing could result in gender bias in IADL prevalence |
| Study size | 10 | Explain how the study size was arrived at | 8 | SABE-COL asked detailed questions of difficulty performing ADLs, IADLs and mobility limitations. Of the 23,694 respondents in SABE, we excluded 68 cases that had missing data on disability status. Our analytic sample consisted of 23,619 respondents. |

Continued on next page

| Quantitative variables | 11 | Explain how quantitative variables were handled in the analyses. If applicable, describe which groupings were chosen and why | 10 | This method requires age-and-sex-specific information on prevalence of disability and mortality rates. With this information we constructed gender specific life tables, and we divide the number of person years lived in a given age interval (from period life table) into years lived with and without disability. We used sampling weights to adjust for differential sampling probability and non-response, when calculating prevalence rates. In addition to gender-specific life tables, we constructed life tables for three measures of disability: ADL, IADL and mobility disability. |
| --- | --- | --- | --- | --- |
| Statistical methods | 12 | (*a*) Describe all statistical methods, including those used to control for confounding | 10 | We used the Sullivan method to calculate prevalence-based life expectancies, which provide the average number of years lived in good and poor health. |
|  |  | (*b*) Describe any methods used to examine subgroups and interactions | N/A | N/A |
|  |  | (*c*) Explain how missing data were addressed | 8 | We excluded 68 cases that had missing data on disability status. |
|  |  | (*d*) *Cohort study*—If applicable, explain how loss to follow-up was addressed  *Case-control study*—If applicable, explain how matching of cases and controls was addressed  *Cross-sectional study*—If applicable, describe analytical methods taking account of sampling strategy | N/A | N/A |
|  |  | (*e*) Describe any sensitivity analyses | N/A | N/A |
| Results | | | | |
| Participants | 13* | (a) Report numbers of individuals at each stage of study—eg numbers potentially eligible, examined for eligibility, confirmed eligible, included in the study, completing follow-up, and analysed | N/A | N/A |
|  |  | (b) Give reasons for non-participation at each stage | N/A | N/A |
|  |  | (c) Consider use of a flow diagram | N/A | N/A |
| Descriptive data | 14* | (a) Give characteristics of study participants (eg demographic, clinical, social) and information on exposures and potential confounders | N/A | N/A |
|  |  | (b) Indicate number of participants with missing data for each variable of interest | 8 | Of the 23,694 respondents in SABE, we excluded 68 cases that had missing data on disability status. |
|  |  | (c) *Cohort study*—Summarise follow-up time (eg, average and total amount) | N/A | N/A |
| Outcome data | 15* | *Cohort study*—Report numbers of outcome events or summary measures over time | N/A | N/A |
|  |  | *Case-control study—*Report numbers in each exposure category, or summary measures of exposure | N/A | N/A |
|  |  | *Cross-sectional study—*Report numbers of outcome events or summary measures | 10 | Prevalence of IADL, ADL and Mobility disability by age and gender |
| Main results | 16 | (*a*) Give unadjusted estimates and, if applicable, confounder-adjusted estimates and their precision (eg, 95% confidence interval). Make clear which confounders were adjusted for and why they were included | 11 and 12 | Table 1. Prevalence of IADL, ADL, and mobility disability by age and gender with associated 95% confidence intervals and tests of gender differences, SABE-Colombia 2015  Table 2. Disabled life expectancy (DLE) by age and gender with associated 95% confidence intervals and tests for gender difference, SABE-Colombia 2015. |
|  |  | (*b*) Report category boundaries when continuous variables were categorized | N/A | N/A |
|  |  | (*c*) If relevant, consider translating estimates of relative risk into absolute risk for a meaningful time period | N/A | N/A |

Continued on next page

| Other analyses | 17 | Report other analyses done—eg analyses of subgroups and interactions, and sensitivity analyses | N/A | N/A |
| --- | --- | --- | --- | --- |
| Discussion | | | | |
| Key results | 18 | Summarise key results with reference to study objectives | 16 | This study presents novel estimates of disability prevalence and disabled life expectancy for men and women ages 60 and older in Colombia. IADL and mobility disability rose steeply with age in Colombian men and women starting at age 60, with increases in ADL disability observed from age 75. Compared to men, women have higher prevalence of IADL and mobility disability at all ages, and higher prevalence of ADL disability beginning around age 70. Among Colombians in their 60s, about one-third (men) to one-half (women) of remaining life will be spent with an IADL or mobility disability. By age 85 nearly all remaining life will be spent with IADL disability and well over half will be spent with mobility disability. |
| Limitations | 19 | Discuss limitations of the study, taking into account sources of potential bias or imprecision. Discuss both direction and magnitude of any potential bias | 21 | This study has some limitations. First, given that the data that we are using is cross sectional, we could not take into account transitions across disabled states (i.e., recovery from disability). Therefore, our analysis is relying on a strong assumption that once in a disabled state the individual continues in that state. There are currently no population-based longitudinal surveys of older adults in Colombia from which disability transitions can be calculated. Additionally, SABE-Colombia surveyed only community-dwelling older adults and thus did not include the institutionalized older adult population, who tend to have the greatest care needs. |
| Interpretation | 20 | Give a cautious overall interpretation of results considering objectives, limitations, multiplicity of analyses, results from similar studies, and other relevant evidence | 16-22 |  |
| Generalisability | 21 | Discuss the generalisability (external validity) of the study results | 16 | This study presents novel estimates of disability prevalence and disabled life expectancy for men and women ages 60 and older in Colombia. |
| Other information | |  | | |
| Funding | 22 | Give the source of funding and the role of the funders for the present study and, if applicable, for the original study on which the present article is based | 22 | Research reported in this publication was supported by the National Institute On Aging of the National Institutes of Health under Award Number T32AG000037 |

*Give information separately for cases and controls in case-control studies and, if applicable, for exposed and unexposed groups in cohort and cross-sectional studies.

**Note:** An Explanation and Elaboration article discusses each checklist item and gives methodological background and published examples of transparent reporting. The STROBE checklist is best used in conjunction with this article (freely available on the Web sites of PLoS Medicine at http://www.plosmedicine.org/, Annals of Internal Medicine at http://www.annals.org/, and Epidemiology at http://www.epidem.com/). Information on the STROBE Initiative is available at www.strobe-statement.org.
