# Supplementary material for: Disabled life expectancy among older Colombian men and women
Source: PLoS One. 2024 Jan 11;19(1):e0296638. doi: 10.1371/journal.pone.0296638 (PMC10783758; doi:10.1371/journal.pone.0296638)
Supplement: S1 Table — (DOCX) [file pone.0296638.s002.docx]

# Supporting Information

| Supporting table 1. Years lived with and without disability, proportion of years disabled and life expectancy for both men and women by age categories | | | | | | | | | | | | | | | |  |
| --- | --- | --- | --- | --- | --- | --- | --- | --- | --- | --- | --- | --- | --- | --- | --- | --- |
|  | **Age Category** |  |  | **Men** | | |  |  |  | **Women** | | |  |  |  | |
|  |  | **LE** |  | Years | | Proportion of years disabled | (95% CI) |  | **LE** | Years | | Proportion of years disabled | (95% CI) |  | P Values | |
|  |  |  |  | Disabled | Not Disabled |  |  |  |  | Disabled | Not Disabled |  |  |  |  |  |
| **IADLs** | 60-64 | 20.64 |  | 6.69 | 13.95 | 32.43 | (6.52 , 6.86 ) |  | 23.6 | 10.94 | 12.62 | 46.43 | (10.76 , 11.11 ) |  | <0.001 | |
|  | 65-69 | 16.96 |  | 6.52 | 10.44 | 38.44 | (6.35 , 6.69 ) |  | 19.4 | 10.36 | 9.09 | 53.25 | (10.19 , 10.53 ) |  | <0.001 | |
|  | 70-74 | 13.67 |  | 6.39 | 7.28 | 46.75 | (6.22 , 6.56 ) |  | 15.7 | 9.81 | 5.85 | 62.64 | (9.65 , 9.98 ) |  | <0.001 | |
|  | 75-79 | 10.82 |  | 6.15 | 4.67 | 56.83 | (5.97 , 6.33 ) |  | 12.3 | 8.92 | 3.38 | 72.49 | (8.77 , 9.07 ) |  | <0.001 | |
|  | 80-84 | 8.46 |  | 5.88 | 2.58 | 69.54 | (5.70 , 6.07 ) |  | 9.4 | 7.83 | 1.62 | 82.86 | (7.69 , 7.96 ) |  | <0.001 | |
|  | 85+ | 6.60 |  | 5.42 | 1.18 | 82.09 | (5.23 , 5.6 ) |  | 7.2 | 6.67 | 0.50 | 93.07 | (6.55 , 6.78 ) |  | <0.001 | |
| **ADLs** | 60-64 | 20.64 |  | 2.44 | 18.20 | 11.81 | (3.99 , 4.3 ) |  | 23.6 | 4.14 | 19.41 | 17.59 | (2.30 , 2.57 ) |  | <0.001 | |
|  | 65-69 | 16.96 |  | 2.37 | 14.59 | 13.97 | (3.89 , 4.21 ) |  | 19.4 | 4.05 | 15.40 | 20.83 | (2.23 , 2.51 ) |  | <0.001 | |
|  | 70-74 | 13.67 |  | 2.40 | 11.27 | 17.57 | (3.86 , 4.19 ) |  | 15.7 | 4.03 | 11.64 | 25.72 | (2.26 , 2.55 ) |  | <0.001 | |
|  | 75-79 | 10.82 |  | 2.52 | 8.31 | 23.26 | (3.90 , 4.26 ) |  | 12.3 | 4.08 | 8.22 | 33.15 | (2.36 , 2.68 ) |  | <0.001 | |
|  | 80-84 | 8.46 |  | 2.55 | 5.91 | 30.12 | (3.84 , 4.23 ) |  | 9.4 | 4.04 | 5.41 | 42.75 | (2.36 , 2.74 ) |  | <0.001 | |
|  | 85+ | 6.60 |  | 2.69 | 3.90 | 40.83 | (3.55 , 4.00 ) |  | 7.2 | 3.77 | 3.39 | 52.67 | (2.46 , 2.93 ) |  | <0.001 | |
| **Mobility Disability** | 60-64 | 20.6 |  | 6.40 | 14.24 | 31.01 | (11.54 , 11.94 ) |  | 23.6 | 11.73 | 11.83 | 49.79 | (11.91 , 6.58 ) |  | <0.001 | |
|  | 65-69 | 17.0 |  | 6.03 | 10.93 | 35.53 | (10.58 , 10.96 ) |  | 19.4 | 10.75 | 8.69 | 55.29 | (10.93 , 6.20 ) |  | <0.001 | |
|  | 70-74 | 13.7 |  | 5.69 | 7.98 | 41.63 | (9.68 , 10.05 ) |  | 15.7 | 9.86 | 5.81 | 62.91 | (10.03 , 5.87 ) |  | <0.001 | |
|  | 75-79 | 10.8 |  | 5.46 | 5.36 | 50.46 | (8.55 , 8.90 ) |  | 12.3 | 8.71 | 3.59 | 70.81 | (8.87 , 5.65 ) |  | <0.001 | |
|  | 80-84 | 8.5 |  | 4.91 | 3.55 | 58.04 | (7.40 , 7.73 ) |  | 9.4 | 7.55 | 1.89 | 79.95 | (7.71 , 5.11 ) |  | <0.001 | |
|  | 85+ | 6.6 |  | 4.59 | 2.01 | 69.50 | (5.96 , 6.30 ) |  | 7.2 | 6.11 | 1.05 | 85.36 | (6.27 , 4.81 ) |  | <0.001 | |
